# Overexpression of glycosyltransferase 8 domain containing 2 confers ovarian cancer to CDDP resistance by activating FGFR/PI3K signalling axis

**DOI:** 10.1038/s41389-021-00343-w

**Published:** 2021-07-22

**Authors:** Shuting Huang, Suiying Liang, Guandi Chen, Jing Chen, Keli You, Haiyan Ye, Zhigang Li, Shanyang He

**Affiliations:** 1 Department of Gynecology, Guangdong Provincial People’s Hospital, Guangdong Academy of Medical Sciences, Guangzhou, 510080 Guangdong P. R. China; 2 The Second School of Clinical Medicine, Southern Medical University, Guangzhou, 510510 Guangdong China; 3 Guangdong Provincial People’s Hospital, School of Medical, South China University of Technology, Guangzhou, Guangdong P. R. China

**Keywords:** Gynaecological cancer, Cell growth, Apoptosis, Oncogenes

## Abstract

It has been reported that chemotherapy resistance mainly contributed to treatment failure and poor survival in patients with ovarian cancer. Therefore, clarifying the molecular mechanism and identifying effective strategies to overcome drug resistance may play an important clinical impact on this malignant tumor. In our study, we found that the expression of Glycosyltransferase 8 domain containing 2 (GLT8D2) was significantly upregulated in ovarian cancer samples with CDDP (Cis-dichlorodiammine-platinum) resistance. Biological experiment demonstrate that GLT8D2 overexpression confers CDDP resistance on ovarian cancer cells; however, inhibition of GLT8D2 sensitized ovarian cancer cell lines to CDDP cytotoxicity both in vitro and in vivo. By using affinity purification/mass spectrometry (IP/MS) and reciprocal co-immunoprecipitation (co-IP) analyses, we found that GLT8D2 interacts with fibroblast growth factor receptor 1(FGFR1) in ovarian cancer cells. Furthermore, overexpression of GLT8D2 activated FGFR/PI3K signaling axis and upregulated the phosphorylation levels of FRS2a and AKT (AKT serine/threonine kinase). Importantly, pharmacological inhibition of FGFR and PI3K (phosphatidylinositol 3-kinase) signaling pathway significantly counteracted GLT8D2-induced chemoresistance and enhanced platinum’s therapeutic efficacy in ovarian cancer. Therefore, our findings suggest that GLT8D2 is a potential therapeutic target for the treatment of ovarian cancer; targeting GLT8D2/FGFR/PI3K/AKT signaling axis may represent a promising strategy to enhance platinum response in patients with chemoresistant ovarian cancer.

## Introduction

Ovarian cancer, which accounts for 4% of all cancers in women, is the second common cause of gynaecologic cancer death in women worldwide [1, 2]. The outcomes of ovarian cancer patients are dim and complicated because of diagnosed late and composed of several subtypes with complex, distinct biological properties [3]. Upfront treatment of ovarian cancer largely relies on debulking surgery followed by platinum–paclitaxel combination chemotherapy [4, 5]. However, but up to 75% of patients will relapse and most will develop a drug-resistant disease, finally the overall 5-year survival rate is just 20% [6, 7]. Therefore, elucidation of the molecular mechanisms underlying chemotherapy resistance and tumor relapse of ovarian cancer is urge to improve clinical outcomes.

Fibroblast growth factor receptors (FGFRs) family is composed of four highly conserved transmembrane receptor with a cytoplasmic tyrosine kinase domain and one receptor has the ability to bind fibroblast growth factor (FGF) ligands [8, 9]. Fibroblast growth factor receptors activation by FGFs initiates a series of intracellular events that regulates cellular differentiation, survival and proliferative signaling pathways [10–12]. Deregulation of this pathway play an important role in various cancers from malignant transformation and tumor development, and therefore be responsible for the emergence of the hallmarks of cancer. Reis-Filho et al. reported that FGFR1 overexpression was detected in more than 40% of classic lobular carcinomas, a subtype of invasive breast cancers [13]. In vitro and in vivo analysis shown that abolishment of FGFR1 signalling inhibited cell viability, however overexpression of FGFR1 shown invasive cell behaviors mainly via induction deregulation of matrix metalloproteinase 3 and E-cadherin [13, 14]. Moreover, aberrant expression of FGFR1 is detected in ALDH^high^ (ALDH + ) pancreatic cancer cells and enhance the tumorigenesis and CSCs(cell stem cell)-like phenotype of PDAC (pancreatic ductal adenocarcinoma) cells with increased expression of Oct4 (POU class 5 homeobox 1), Sox-2 (SRY-box transcription factor 2), Nanog (Nanog homeobox), and c-Myc (MYC proto-oncogene, bHLH transcription factor), but targeting FGFR signalling with the selective FGFR1 inhibitor, PD173074 inhibited the proliferation and self-renewal of the panCSCs [15]. The above studies suggest that FGFR signalling play an important role in tumor development and targeting it may be increasing the efficacy of therapies with the traditional chemotherapy drugs.

Glycosyltransferases are a large class of enzymes that transfer one or more sugar molecules to a series of receptor molecules, such as lipids, proteins, hormones, secondary metabolites, and oligosaccharides [16–18]. It has been reported that glycosyltransferases play critical roles in regulating several basic biologic processes, including tissue development, cell signaling, cellular adhesion and carcinogenesis [19–22]. Glycosyltransferase 8 domain containing 2 (GLT8D2) is a novel glycosyltransferase which was located in 12q23.3 and broad expression in gall bladder (RPKM 16.0) and ovary (RPKM 13.7). GLT8D2 was firstly cloned from HepG2 cells and interacted with apoB100, and positively regulated the levels of apoB100 protein via glycosyltransferase catalyses apoB100 glycosylation in HepG2 cells [23]. Zhan et al. subsequently demonstrate that GLT8D2 participated in non-alcoholic fatty liver disease (NAFLD) pathogenesis via negatively regulating microsomal triglyceride transfer protein (MTP) in HepG2 cells [24]. These studies suggesting a pivotal role in regulation of liver disease, however, the biological function and precise molecular mechanism of GLT8D2 in tumor development, especially in ovarian cancer chemoresistance remain unclear.

## Materials and methods

### Chemical reagents

CDDP were purchased from Sigma-Aldrich (Germany), and FGFR inhibitor (TAS-120) or AKT inhibitor (GDC-0068) were purchased from Selleck Chemicals.

### Cell lines and cell culture

The ovarian cancer cell lines, including SKOV3, CAOV4, CAOV3, OVCAR, A2780, TOV21G, TOV112D and OV90 was obtained from American Type Culture Collection and cultured in the specific medium according to the manufacturer’s instructions, at 37 °C in a 5% CO2 atmosphere in a humidified incubator. All cell lines were authenticated by short tandem repeat (STR) fingerprinting.

### Patient information and tissue specimens

A total of 39 paraffin-embedded and archived ovarian cancer samples, which were histopathologically and clinically diagnosed at Guangdong Provincial People’s Hospital, were examined in this study. Clinical information on the samples is summarized in Supplementary Table S1. All patients received standard platinum-based chemotherapy. Chemoresistance or chemosensitivity was defined as relapse or progression within 6 months or after 6 months from the last chemotherapy, respectively. Prior patient consent and approval from the Institutional Research Ethics Committee were obtained for the use of these clinical materials for research purposes.

### Vectors, retroviral infection and transfection

GLT8D2 expression construct was generated by subcloning PCR-amplied full-length human GLT8D2 cDNA into the pSin-EF2-puro-flag plasmid, and human GLT8D2-targeting short hairpin RNA (shRNA) oligonucleotides sequences were cloned into pSuper-retro-puro to generate pSuper-retro-shGLT8D2#1 and pSuper-retro-shGLT8D2#2. The shRNA sequences were synthesized by Invitrogen. Transfection of shRNA or plasmids was performed using the Lipofectamine 3000 reagent (Invitrogen, Carlsbad, CA) according to the manufacturer’s instruction. Stable cell lines expressing GLT8D2 or GLT8D2 shRNA were selected for 10 days with 0.5 μg/ml puromycin 48 h after infection.

### Western blot analysis

Western blot was performed using anti-GLT8D2 (Abcam), anti-p-SRS2a, SRS2a and anti-p-AKT, anti-AKT, anti-p-GSK3β, anti-GSK3β, anti-XIAP antibodies (Cell Signaling, Danvers, MA, USA). The membranes were stripped and re-probed with an anti-GADPH antibody (Sigma, Saint Louis, MI) as a loading control.

### Xenografted tumor model and TUNEL staining

In the subcutaneous tumor model, the BALB/c nude mice were randomly divided into four groups (*n* = 5/group). Mice were inoculated subcutaneously with 2 × 10^6^ SKOV3-shRNA-Vector, SKOV3-shRNA#1 cells, SKOV3-shRNA#2 cells respectively, in the left dorsal flank per mouse. After xenografts reached 0.5 cm in diameter, CDDP (5 mg/kg) was given intraperitoneally every 4 days for 28 days. Tumor growth was monitored by measurements of length and width and the tumor volume was calculated using the equation (L × W2)/2. TUNEL assay was performed on paraffin-embedded tissue section according to the manufacturer’s instructions (Promega). Apoptotic index was measured by percentage of TUNEL-positive cells.

### Cytotoxicity assay

The sensitivity to cisplatin of ovarian cancer cells was determined using the MTT assay as previously described. Briefly, 2 × 10^3^ cells were seeded onto 96-well plates and incubated at 37 °C overnight. Cells were then transfected with different concentrations of cisplatin (0–32 μM). After incubation for 48 h, 50 μl of the MTT solution (0.15%) was added to each well, and the plates were further incubated for 2 h. One hundred microliters of DMSO was added to solubilize the MTT formazan product. Absorbance at 540 nm was measured with a Falcon microplate reader (BD-Labware). Dose-response curves were plotted on a semilog scale as the percentage of the control cell number, which was obtained from the sample with no drug exposure. IC50 was determined by the intersection of the cisplatin concentration and the midpoint of the 570 nm reading.

### Apoptosis assay

For evaluation of apoptosis, PE Annexin V Apoptosis Detection Kit I (BD Pharmingen) was used. Briefly, 1 × 10^6^ ovarian cancer cells were plated in 10 cm plates and incubated for 24 h. Treatment was started with cisplatin (12 μM) for 24 h. Cell morphology was assessed by phase-contrast microscopy. Then, cells were removed from plate by trypsin-EDTA, washed twice with PBS, and re-suspended with binding buffer at 10^6^ cells/ml. FITC Annexin V and propidium iodide were added (each at 5 μl/10^5^ cells). Cells were incubated for 15 min at room temperature in the dark. Percentage of apoptosis was analyzed with an EPICS XL flow cytometer (Beckman-Coulter). Each sample was analyzed in triplicate.

### Transient luciferase assay

Cells (1 × 10^4^) were seeded in triplicate in 48-well plates and allowed to settle for 24 h. For each transfection, one hundred nanograms of luciferase reporter plasmids pGL-3-GLT8D2 or vector and 5 ng of pRL-TK, expressing Renilla luciferase as an internal control, were transfected into cells using the Lipofectamine 3000 reagent (Invitrogen) according to the manufacturer’s instruction. 48 h after transfection, cells were harvested and Luciferase and renilla signals were measured using the Dual Luciferase Reporter Assay Kit (Promega) according to a protocol provided by the manufacturer. The luciferase activity was normalized by the Renilla luciferase activity of each transfection to normalize the transfection efficiency.

### Colony formation assay

Cells which treatment with cisplatin (12 μM) were plated on a 6-well plate (0.5 × 10^3^ cells per well) and cultured for 10 days. The colonies were stained with 1.0% crystal violet for 1 min after fixation with 10% formaldehyde for 5 min. The experiment was performed independently three times for each cell line.

### Statistical analysis

Statistical tests for data analysis included Fisher’s exact test, log-rank test, Chi-square test, and Student’s 2-tailed *t* test. Multivariate statistical analysis was performed using a Cox regression model. Statistical analyses were performed using the SPSS 21.0 statistical software package. Data represent mean ± SD. *P* < 0.05 was considered statistically significant.

### Microarray data process and visualization

Microarray data were downloaded from the GEO database and The Cancer Genome Atlas (TCGA) database:

https://www.cancer.gov/about-nci/organization/ccg/research/structural-genomics/tcga; http://www.ncbi.nlm.nih.gov/geo/

GSEA was performed using GSEA 2.0.9: (http://www.broadinstitute.org/gsea/).

## Results

### GLT8D2 is overexpression in human ovarian cancer with chemoresistance and correlates with progression and poor prognosis

**Fig. 1:**
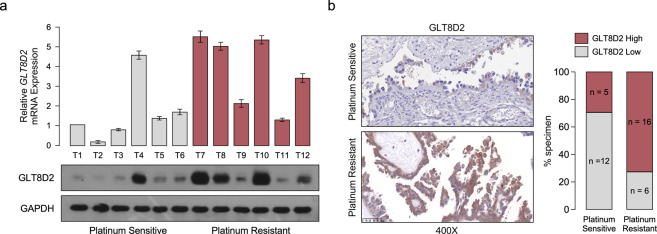
GLT8D2 is overexpression in human ovarian cancer with chemoresistance and correlates with progression and poor prognosis. **a** The mRNAs expression of GLT8D2 in ovarian cancer tissues (up); Western blotting analysis of GLT8D2 expression in 12 human ovarian cancer tissues, GAPDH was used as a loading control. **b** IHC staining indicating the GLT8D2 protein expression in platinum sensitive and platinum resistance tumor tissues.

By analyzing the two published mRNA expression profiles with chemotherapy treatment in ovarian cancer (GSE 51373, MTAB-7083) obtained from Array Express—functional genomics data (https://www.ebi.ac.uk/arrayexpress/), we found that GLT8D2 mRNA was the most significantly upregulated in chemotherapy resistance and platinum resistance tissues in the both published mRNA expression profiles (Supplementary Fig. 1a, b). Furthermore, compare to platinum sensitive tissues, the protein level of GLT8D2 was elevated in the ovarian cancer samples with platinum resistance by performing western blotting and IHC analyses (Fig. 1a, b, Supplementary Tables S1). Moreover, overexpression of GLT8D2 was associated with chemoresistance in human ovarian cancer, which show that 72.7% of GLT8D2-overexpression ovarian cancer samples cases exhibited chemoresistance (Fig. 1b), suggesting that GLT8D2-overexpression might be involved in ovarian cancer chemotherapy failure. Consistently, analysis of TCGA datasets showed that overexpression of GLT8D2 was significantly associated with ovarian cancer poor survival by Gene Set Enrichment Analysis (GSEA) (Supplementary Fig. 1c). Furthermore, GLT8D2 was also significantly increased in ovarian cancer and was positively correlated with shorter overall, relapse-free survival and post progression survival in ovarian cancer patients with platinum-based chemotherapy (Supplementary Fig. 1d). By using the stepwise regression in Univariate analysis and multivariate Cox regression models, we identified the variables of chemotherapy outcome (HR = 2.722(1.743–4.249), *p* = 0) and GLT8D2 expression (HR = 1.474(1.027–2.115), *p* = 0.035) as an independent prognostic factor for overall survival of ovarian cancer patients, but not Age, Clinical Stage (Supplementary Tables S1, S3). Taken together, our results suggest that overexpression of GLT8D2 is involved in chemoresistance and correlates with progression and poor prognosis in human ovarian cancer.

### Upregulation of GLT8D2 confers CDDP resistance in ovarian cancer in vitro

**Fig. 2:**
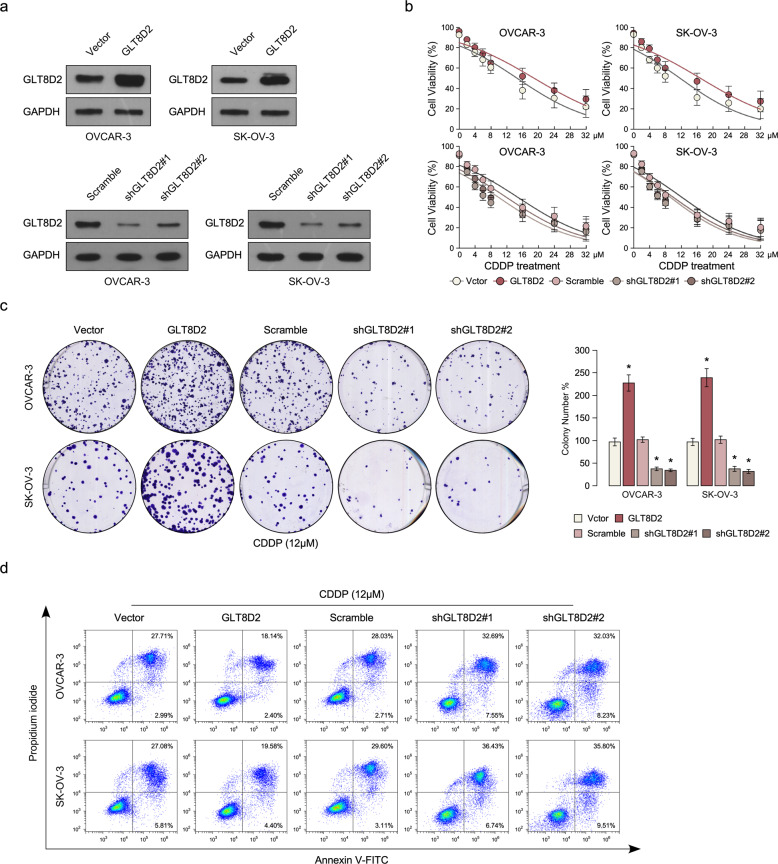
Upregulation of GLT8D2 confers CDDP resistance in ovarian cancer in vitro. **a** Western blotting analysis of the protein expression levels of GLT8D2 in the indicated cells. GADPH was used as a loading control. **b** Cell viability in the indicated cells. **c** Quantification of the number of colonies formed by the indicated ovarian cancer cells. * *P* < 0.05. **d** Annexin V-FITC and PI staining of the indicated cells treated with cisplatin (10 μM) for 24 h. Each bar represents the mean ± SD of three independent experiments.

To investigate the chemoresistance role of GLT8D2 in ovarian cancer progression, we firstly examine the protein expression of GLT8D2 in ovarian cancer cell lines and OVCAR3 and SKOV3 cancer cell lines was chose for stably overexpressed and knockout GLT8D2 expression (Fig. 2a and Supplementary Fig. 2b). Cell viability assay show that overexpression of GLT8D2 enhanced CDDP resistance compared with the vector-transfected cells, however, inhibition of GLT8D2 in ovarian cancer cells were more sensitive to CDDP treatment than control-transfected cells (Fig. 2b). Colony formation assay show that overexpression of GLT8D2 significantly enhanced the anti-apoptosis ability compared with the vector control, however, inhibition of GLT8D2 in ovarian cancer cells reverse it (Fig. 2c). Furthermore, Annexin V assay show that the percentage of apoptotic cells in GLT8D2-overexpression ovarian cancer cells treated with CDDP was much lower compared than that in control cells, but much higher in GLT8D2-silenced cells (Fig. 2d). The above results indicating that deregulation of GLT8D2 is involved in CDDP resistance of ovarian cancer cells in vitro.

### Inhibition of GLT8D2 sensitize ovarian cancer to CDDP treatment in in vivo

**Fig. 3:**
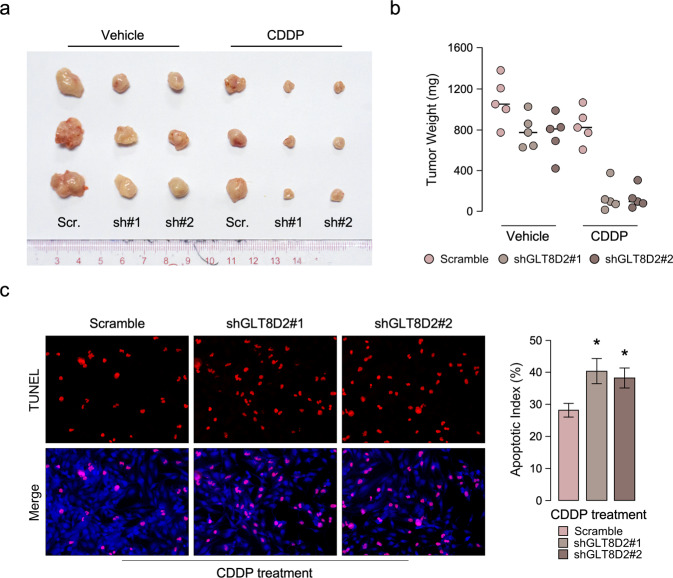
Inhibition of GLT8D2 sensitize ovarian cancer to CDDP treatment in in vivo. **a** Tumor bearing in the indicate mice. **b** Tumor weights of the subcutaneous tumor model in indicated mice. **c** IHC staining demonstrated the expression of TUNEL-positive cells in the indicated tissues, *P < 0.05.

In order to explore the function of GLT8D2 in ovarian cancer chemoresistance, the in vivo subcutaneous inoculation models to assess the anti-CDDP effect of GLT8D2 in ovarian cancer. Nude mice were subcutaneously inoculated with SKOV3-Scr, SKOV3-GLT8D2 –shRNA#1, SKOV3-GLT8D2 –shRNA#2 respectively, mouse were treated with CDDP or vehicle when the treatment with drugs started as soon as the tumor became palpable. As shown in Fig. 3a, b, compare to the vehicle treatment, GLT8D2-shRNA plus CDDP treatment resulted in a significantly reduction in tumor growth and tumor weight compared with that in the control group. Consistently, immunofluorescence assay showed that inhibition of GLT8D2 conferred great sensitive to chemotherapy-induced apoptosis, as determined by proportion of TUNEL^+^-cells compared with that in the control group (Fig. 3c). Therefore, these results demonstrated that inhibition of GLT8D2 sensitize cancer cells to CDDP treatment in ovarian cancer.

### Upregulation of GLT8D2 activates the FGFR/PI3K signalling axis in ovarian cancer

**Fig. 4:**
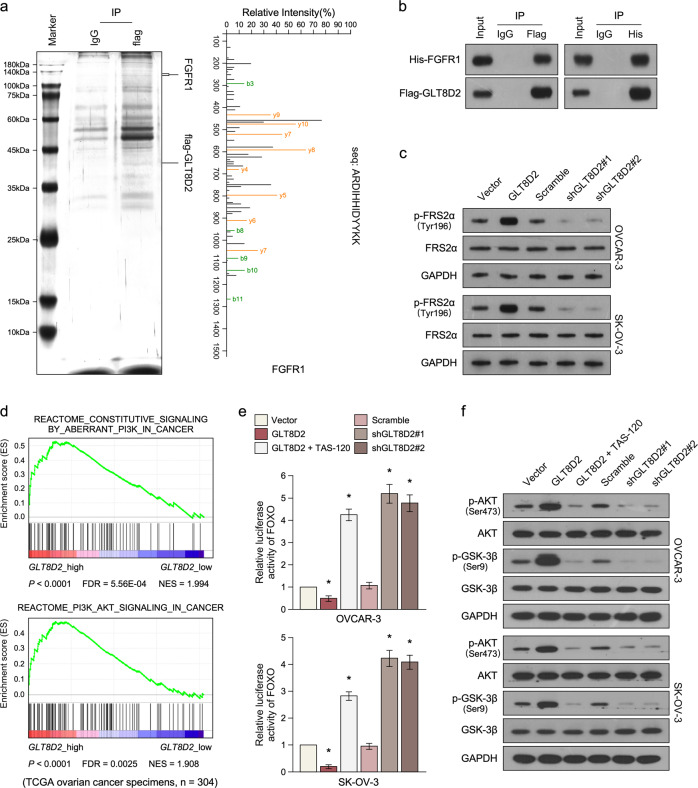
Upregulation of GLT8D2 activates the FGFR/PI3K signalling axis in ovarian cancer. **a** Lysates from Flag- GLT8D2 overexpression cancer cells were immunoprecipitated using anti-Flag affinity agarose, followed by mass-spectrometric peptide sequencing. **b** Co-IP assay showing that GLT8D2 interacted with FGFR1. **c** Western blotting analysis of the phosphorylation levels of FRS2a and AKT in the indicated cells. GADPH was used as a loading control. **d** GSEA analysis showing that GLT8D2 expression was correlated with PI3K/AKT target gene signatures in TCGA ovarian cancer datasets. **e** Analysis of luciferase reporter activity in the indicated cells after transfection with 100 ng FOXO-luciferase plasmids or control-luciferase plasmid, * *P* < 0.05. **f** Western blotting analysis of the expression levels the phosphorylation levels of AKT and GSK3b in the indicated cells. GADPH was used as a loading control.

In order to better understand the mechanism underlying GLT8D2 overexpressed induced chemoresistance, the potent GLT8D2-binding proteins was identified by immunoprecipitation/mass spectrometry (IP/MS). As show in Fig. 4a, b, IP/MS and reciprocal co-immunoprecipitation (co-IP) analyses demonstrated that GLT8D2 interacts with fibroblast growth factor receptor 1(FGFR1). Western blot assay shows that phosphorylation levels of FRS2a, which is essential for sustained activation of the protein tyrosine phosphatase Shp2 in response to FGF stimulation, was significantly increase in GLT8D2 overexpression group but decrease GLT8D2-downregulation group (Fig. 4c). Furthermore, PI3K/AKT is an important downstream signalling pathway of FGF, we examine GLT8D2 whether play an effect in PI3K/AKT signalling. Gene Set Enrichment Analysis (GSEA) show that the mRNA levels of GLT8D2 expression in ovarian cancer not only correlation with the FGFR-activated gene signatures but also with PI3K/AKT-activated gene signatures in published datasets (Fig. 4d and Supplementary Fig. 3a). Overexpression of GLT8D2 significantly enhanced, whereas silencing of GLT8D2 reduced, the activity of FOXO luciferase reporter activity in OVCAR-3 and SKOV3 cancer cells (Fig. 4e). Moreover, the activity of FOXO luciferase reporter activity was significantly repressed in GLT8D2 overexpression group treatment with TAS-120 (PMID: 31109923), which is a highly selective, and irreversible FGFR inhibitor (Fig. 4e). Furthermore, overexpression of GLT8D2 significantly increase the phosphatase levels of AKT and GSK3β but decrease levels of the important downstream molecules of PI3K/AKT signalling, such as p21 and p27, and downregulation of GLT8D2 reverse it (Fig. 4f and Supplementary Fig. 3b). Consistently, the increase levels of phosphatase levels of AKT and GSK3β was significantly repressed by using TAS-120 treatment in GLT8D2 overexpression group, and the decrease protein levels of p21 and p27 was significantly reverse by using TAS-120 treatment (Fig. 4f and Supplementary Fig. 3b). Meanwhile, we analyzed the cell cycle in GLT8D2-overexpression and GLT8D2-downregulation cells by flow cytometry, which showed significant increase in the percentage of cells in S phase in GLT8D2-overexpression cells but decrease in GLT8D2-downregulation cells (Supplementary Fig. 3c). Moreover, we found that the increase in the percentage of cells in S phase in GLT8D2-overexpression cells was reversed by treatment with a FGFR inhibitor (Supplementary Fig. 3c). These results suggesting that GLT8D2 plays an important role in activating the FGFR/PI3K signalling pathway in ovarian cancer.

### Clinical relevance of GLT8D2 and FGFR/PI3K signalling in human ovarian cancer

**Fig. 5:**
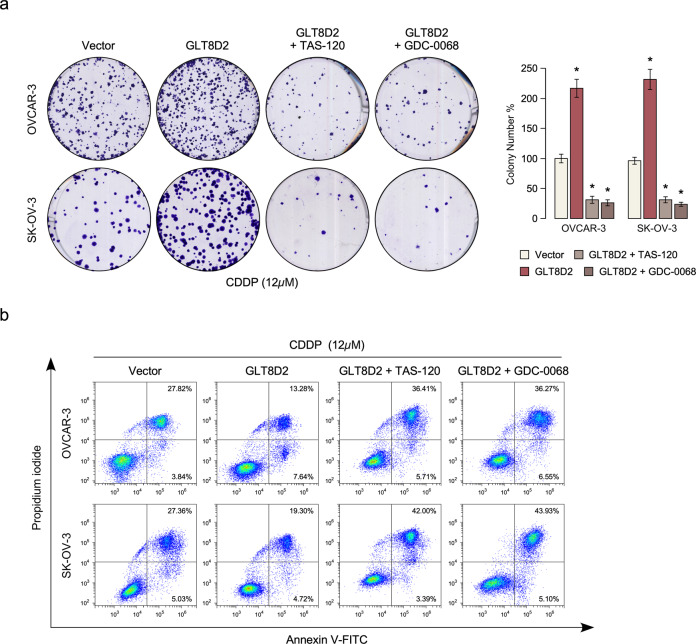
FGFR/PI3K signalling pathway is required for GLT8D2-induced chemoresistance. **a** Quantification of colony numbers in ovarian cancer cells treated with FGFR inhibitor or AKT inhibitor, as determined by colony formation, *P < 0.05. **b** Annexin V-FITC and PI staining of the indicated cells treated with FGFR inhibitor or AKT inhibitor.

**Fig. 6:**
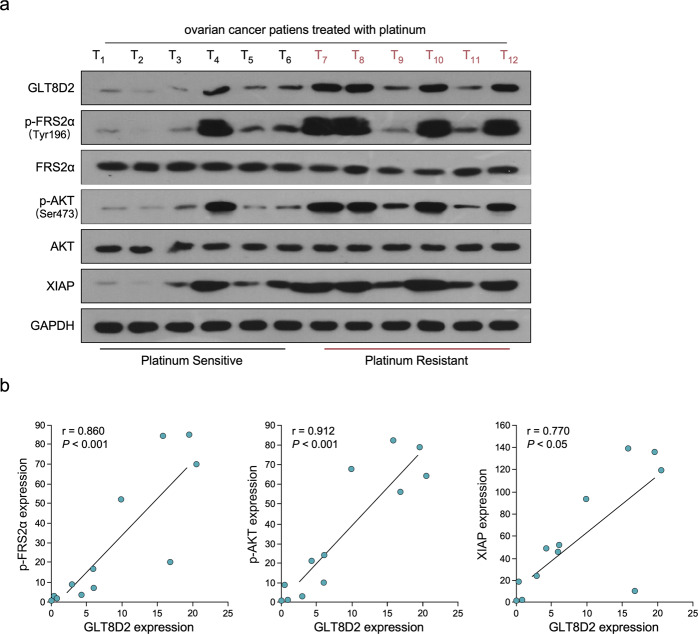
Clinical relevance of GLT8D2-induced FGFR/PI3K activation in human ovarian cancer. **a** Expression analysis the protein expression of GLT8D2, phosphatase levels of FRS2a (Tyr196), AKT (Ser473) and XIAP in 12 freshly collected human ovarian cancer tissue samples (T); GAPDH was used as loading controls. **b** Correlation analysis of GLT8D2 expression and p-FRS2a (Tyr196), p-AKT (Ser473) and XIAP in 12 freshly collected human ovarian cancer tissue samples (T).

Next, we investigated whether GLT8D2-mediated ovarian cancer chemoresistance occurs through FGFR/PI3K activation. As shown in Fig. 5a, b, the chemoresistance effect of GLT8D2 in ovarian cancer cells was significantly inhibited by treatment with a FGFR inhibitor or AKT inhibitor (GDC-0068) (PMID: 23287563). Meanwhile, the clinical relevance of GLT8D2 expression and FGFR/PI3K activation was further characterized in human ovarian cancer. Firstly, we found that the expression of GLT8D2, FRS2a (Tyr196), AKT (Ser473) and XIAP were significantly increase in six platinum resistant clinical ovarian cancer samples, compared with six platinum sensitive clinical ovarian cancer samples (Fig. 6a). As showed in Fig. 6a, b, GLT8D2 levels in 12 freshly collected clinical ovarian cancer samples with chemotherapy were positively correlated with phosphatase levels of FRS2a (Tyr196) (*r* = 0.86, *P* < 0.001) and AKT (Ser473) (*r* = 0.912, *P* < 0.001). The correlation of GLT8D2 expression and XIAP, an important anti-apoptosis factor and downstream molecules of PI3K/AKT signalling, was further confirmed in clinical ovarian cancer samples with chemotherapy (*r* = 0.77, *P* < 0.05; Fig. 6a, b). These data further support the notion that GLT8D2 upregulation confers ovarian cancer chemoresistance and activation of the FGFR/PI3K signalling axis, which may lead to a poor clinical outcome for patients with ovarian cancer.

## Discussion

In the current study we demonstrate that the expression of glycosyltransferase GLT8D2 was significantly upregulated in CDDP resistance-ovarian cancer samples and GLT8D2 confers CDDP resistance on ovarian cancer cells both in vitro and in vivo. Interestingly, we found that GLT8D2 interacts with FGFR1 in ovarian cancer cells and activated FGFR signaling, subsequently activated the PI3K/AKT signaling pathway. Importantly, pharmacological inhibit both FGFR and PI3K/AKT signaling significantly reversed GLT8D2-induced chemoresistance and enhanced platinum’s therapeutic efficacy in ovarian cancer. Therefore, our findings suggest that GLT8D2 is a potential therapeutic target for the treatment of ovarian cancer.

Abnormal activation of FGF (FGF)/FGFR (fibroblast growth factor receptor) signalling contribute to the promotion of several oncogenic mechanisms: proliferation, anti-apoptosis, cytoskeleton modifications, angiogenesis, and chemoresistance [25–27]. FGF/FGFR signalling activation can initiate a series of downstream pathways, such as PI3K/AKT signaling to sustain cellular proliferation, differentiation, and survival. Katarzyna etc. reported that FGFR1 and 3 expression is associated with regulatory PI3K/AKT kinase activity and promotes invasion and prognosis of human laryngeal cancer [28]. Furthermore, FGF 2 induces proliferation and distribution of G2/M phase of bovine endometrial cells via activating of PI3K/AKT signaling [29]. Therefore, inhibition of FGFR or PI3K/AKT signaling pathway may be more effective in inhibiting tumor growth and may be putative therapeutic target in cancer. Interestingly, in our study we found that the anti-CCP effect of GLT8D2 in ovarian cancer cells was significantly inhibited by treatment with FGFR inhibitor (TAS-120) or AKT inhibitor (GDC-0068) by colony formation and Annexin V-FITC assay. The above studies suggest that targeting FGFR or PI3K/AKT pathway can increase the efficacy of therapies with the traditional chemotherapy drugs in ovarian cancer cells.

The glycosyltransferases family proteins have been reported to be fundamentally involved in regulating several basic biologic processes, such as cell development, cell migration and invasion and carcinogenesis [21, 30–32]. For example, anomalous glycosylation is a hallmark of several cancers, including ovarian cancer, that promote tumor progression and metastasis [33, 34]. Similarity, the glycosyltransferases ST6Gal I reduced the activation of caspase 3 and protected against cell death after cisplatin treatment, which indicates that ST6Gal I may be a novel contributor to cisplatin resistance in ovarian cancer [35]. While aberrant glycosylation patterns have led to tumorigenesis, the underlying molecular mechanisms by which they contribute to tumor progression, however, remain poorly understood. It has previously been reported that GLT8D2 participated in NAFLD pathogenesis via negatively regulating MTP in HepG2 cells [24], but the biological function and molecular mechanism of GLT8D2 in chemoresistance remain unclear. Herein, we found that overexpression of GLT8D2 confers CDDP resistance to ovarian cancer cells via activating the FGFR/PI3K/AKT signaling pathway. Importantly, pharmacological inhibit both FGFR and PI3K/AKT signaling significantly reversed GLT8D2-induced chemoresistance and enhanced platinum’s therapeutic efficacy in ovarian cancer, suggesting that GLT8D2 could contribute to FGFR/PI3K/AKT activation and thereby represent a novel target for ovarian cancer treatment. Previously study has been reported that hyper-actively of PI3K/Akt/GSK signalling pathway may have induced mitochondrial dysfunction and Akt1 is the major isoform that modulated mitochondrial Complex V activity [36]. Yang et al. also showed that activation of mitochondrial Akt1 enhanced ATP production and increased phosphocreatine in cardiac muscle cells but dysregulation of Akt signalling pathway might impair mitochondrial bioenergetics in diabetic myocardium [37]. Therefore, as a member of glycosyltransferases family proteins, it would be interesting to explore whether GLT8D2 enhanced cisplatin resistance in ovarian cancer via regulating mitochondrial oxidative phosphorylation or by regulating the levels of ROS, which will be carried out in our laboratory.

## Supplementary information

Supplementary information

Supplementary Figure 1

Supplementary Figure 2

Supplementary Figure 3
