# Supplementary material for: Overexpression of glycosyltransferase 8 domain containing 2 confers ovarian cancer to CDDP resistance by activating FGFR/PI3K signalling axis
Source: Oncogenesis. 2021 Jul 22;10(7):55. doi: 10.1038/s41389-021-00343-w (PMC8298492; doi:10.1038/s41389-021-00343-w)
Supplement: Supplementary file 1 — Supplementary information [file 41389_2021_343_MOESM1_ESM.doc]

**Supplementary information**

**Supplementary Figure Legends**

**Supplementary Figure 1. GLT8D2 is overexpression in human ovarian cancer with chemoresistance and correlates with progression and poor prognosis.** (a). Analysis the mRNA level of GLT8D2 in public ovarian cancer dataset by Venn Diagram. (b). The mRNA of GLT8D2 was significantly overexpression in public ovarian cancer dataset. (c). GSEA analysis showing that GLT8D2 expression was correlated with poor survival of ovarian cancer. (d). Kaplan-Meier survival analysis of low GLT8D2 protein expression and high expression.

**Supplementary Figure 2.** (a) mRNA (Up) and Western blot (Down) analysis of GLT8D2 in the indicated ovarian cancer cells; GAPDH was used as a loading control. (b) mRNA analysis of GLT8D2 in the indicated ovarian cancer cells. GAPDH was used as a loading control. * *P* <0.05.

**Supplementary Figure 3.** (a) GSEA analysis showing that GLT8D2 expression was correlated with FGFR and PI3K/AKT target gene signatures in TCGA ovarian cancer datasets. (b) Western blotting analysis of the p21, p27 and cyclinD1 in the indicated cells. GADPH was used as a loading control. (c) Flow cytometry analysis of indicated ovarian cancer cells.

**Supplementary Tables**

**Supplementary Tables S1. The relationship between GLT8D2 and clinical pathological characteristics in ovarian cancer patients (n = 39)**

|  |  | **GLT8D2 expression** | |  |
| --- | --- | --- | --- | --- |
| **Parameters** | **Number of cases** | **Low (n = 18)** | **High (n = 21)** | ***P*-value** |
| **Age (years)** |  |  |  |  |
| < 58 | 20 | 10 | 10 | 0.432 |
| ≥ 58 | 19 | 8 | 11 |
| **FIGO stage** |  |  |  |  |
| I /II | 12 | 9 | 3 | 0.019 |
| III / IV | 27 | 9 | 18 |
| **Metastasis** |  |  |  |  |
| Yes | 25 | 12 | 13 | 0.511 |
| No | 14 | 6 | 8 |
| **Chemoresponse status** |  |  |  |  |
| Chemoresistance | 22 | 5 | 17 | 0.010 |
| Chemosensitivity | 17 | 11 | 6 |
| **Recurrence** |  |  |  |  |
| Yes | 26 | 9 | 17 | 0.044 |
| No | 13 | 9 | 4 |

**Supplementary Tables S2. Clinicopathological Characteristics of Studied Patients and Expression of *GLT8D2*** in Ovarian Cancer (TCGA dataset)

| **Characteristics** | **No. of Cases** |
| --- | --- |
| **Age** |  |
| < 55 | 90 |
| ≥ 55 | 141 |
| **Clinical Stage** |  |
| Stage II | 16 |
| Stage III | 188 |
| Stage IV | 27 |
| **Chemotherapy Outcome** |  |
| Complete Response / Partial Response | 193 |
| Stable Disease / Progressive Disease | 38 |
| **Status (at follow-up)** |  |
| Alive | 102 |
| Death because of ovarian cancer | 129 |
| Death because of other than ovarian cancer | 0 |
| ***GLT8D2* expression** |  |
| Low expression | 154 |
| High expression | 77 |

**Supplementary Tables S3.** **Univariate and multivariate analyses of various prognostic parameters in patients with ovarian cancer Cox-regression analysis (TCGA dataset)**

|  | **Univariate analysis** | | | **Multivariate analysis** | |
| --- | --- | --- | --- | --- | --- |
|  | **No. patients** | ***P*** | **Relative risk**  **(95% confidence interval)** | ***P*** | **Relative risk**  **(95% confidence interval)** |
| **Age** |  | | | | |
| < 55 | 90 | 0.124 | 1.335（0.924-1.929） | 0.104 |  |
| ≥ 55 | 141 |  |  |  |  |
| **Chemotherapy Outcome** | | | | | |
| CR/PR | 193 | 0.000 | 2.849(1.828-4.439) | 0.000 | 2.722(1.743-4.249) |
| SD/PD | 38 |  |  |  |  |
| **Clinical Stage** | | | | | |
| Stage II  Stage III  Stage IV | 16  188  27 | 0.615 | 1.107(0.746-1.642) | 0.806 |  |
| **Expression of *GLT8D2*** | | | | | |
| Low expression | 154 | 0.014 | 1.565（1.093-2.241） | 0.035 | 1.474(1.027-2.115) |
| High expression | 77 |  |  |  |  |
