# Supplementary figures and images for: Overexpression of glycosyltransferase 8 domain containing 2 confers ovarian cancer to CDDP resistance by activating FGFR/PI3K signalling axis
Source: Oncogenesis. 2021 Jul 22;10(7):55. doi: 10.1038/s41389-021-00343-w (PMC8298492; doi:10.1038/s41389-021-00343-w)

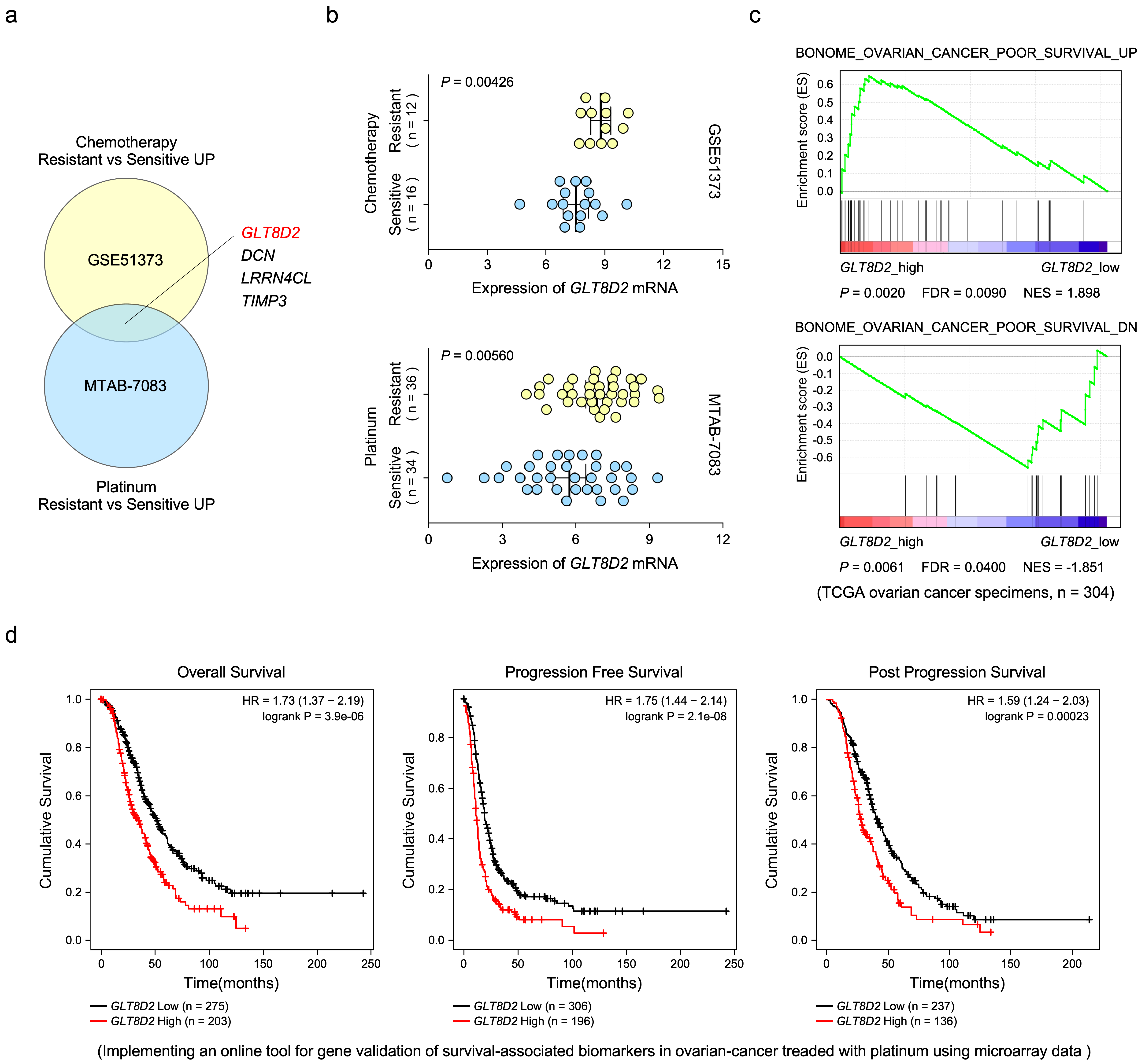

Supplement: Supplementary file 2 — Supplementary Figure 1 [file 41389_2021_343_MOESM2_ESM.tif]

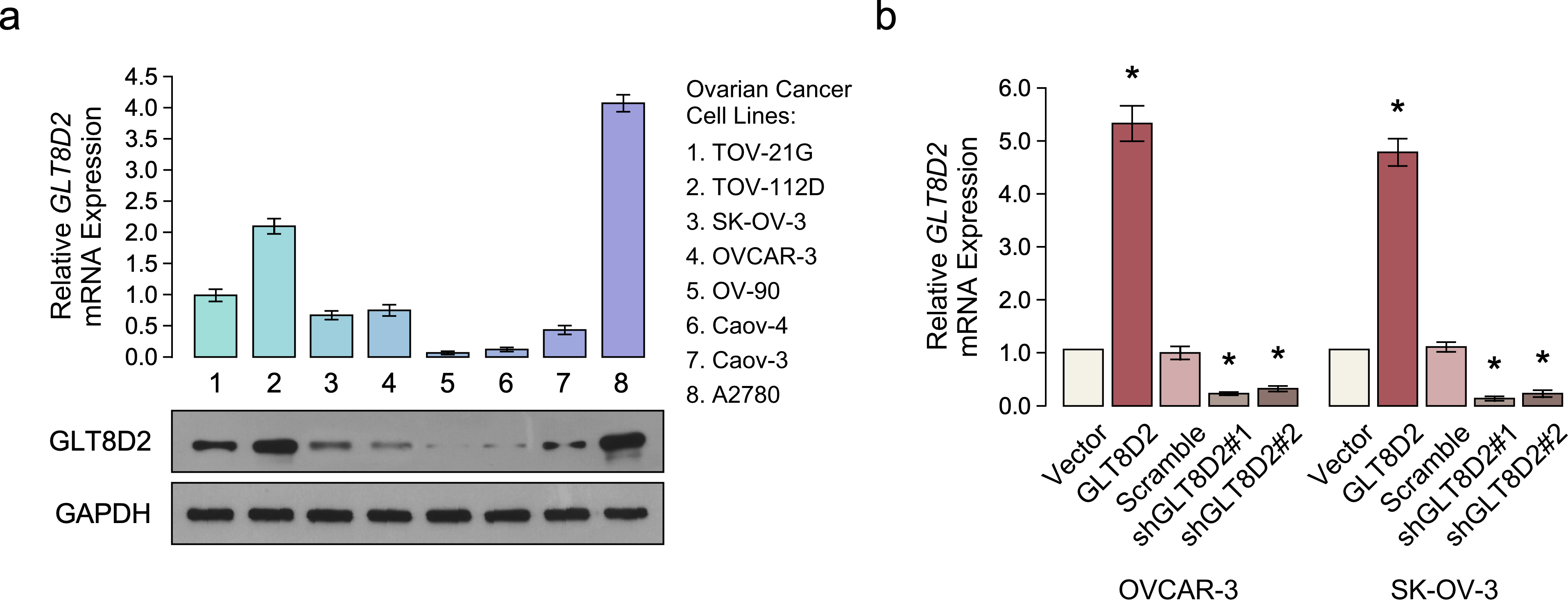

Supplement: Supplementary file 3 — Supplementary Figure 2 [file 41389_2021_343_MOESM3_ESM.tif]

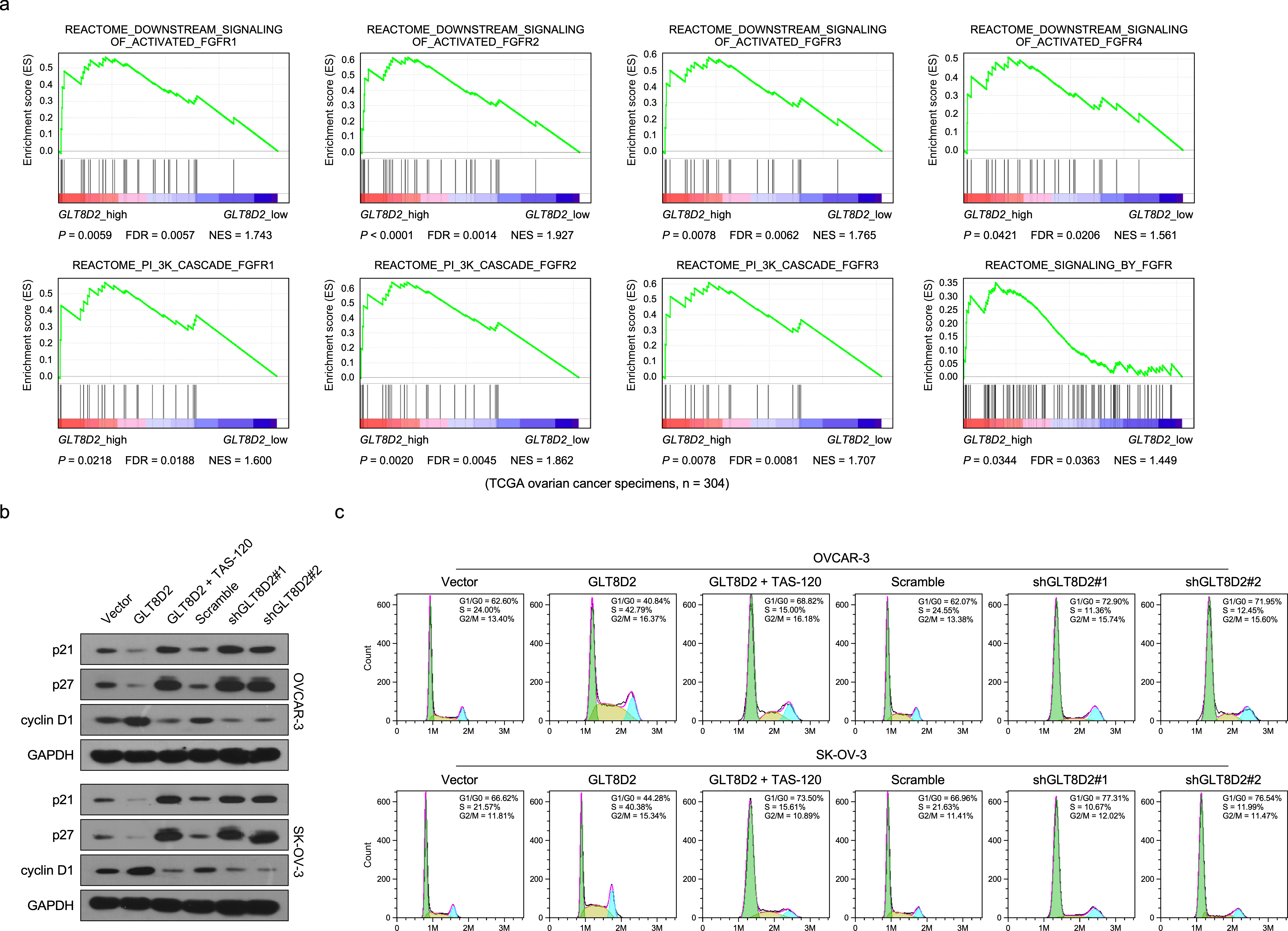

Supplement: Supplementary file 4 — Supplementary Figure 3 [file 41389_2021_343_MOESM4_ESM.tif]
